# Editorial: Microbial roles in caves

**DOI:** 10.3389/fmicb.2024.1411535

**Published:** 2024-05-29

**Authors:** Valme Jurado, Diana E. Northup, Cesareo Saiz-Jimenez

**Affiliations:** ^1^Instituto de Recursos Naturales y Agrobiologia, Consejo Superior de Investigaciones Cientificas (IRNAS-CSIC), Seville, Spain; ^2^Department of Biology, University of New Mexico, Albuquerque, NM, United States

**Keywords:** subsurface microbiology, geomicrobiology, biomineralization, biogeochemical cycles, speleothems, microbiota, *Bacteria*, *Archaea*

Subsurface ecosystems (caves) are a window into hidden niches of the Earth where microorganisms adapt to live in hostile environmental conditions that could be analogs to Mars, particularly volcanic caves and extremely acidic environments. Caves have attracted the attention of NASA and ESA, as the search for life on other Solar System bodies is a major stimulus for planetary exploration. Thus, cave research is useful in astrobiology for searching/identifying extraterrestrial life.

Some caves show high carbon dioxide and radon concentrations throughout the year. In these cases, caves have been considered extreme environments characterized by harsh environmental conditions and low nutrient inputs where microorganisms are forced to adapt their metabolism to survive in extreme conditions, in which the low input of carbon, nitrogen, and phosphorus, as well as the chemical composition of the rock, has a direct impact on the community diversity.

The colonization of cave rocks and speleothems provides complex communities active in the main biogeochemical cycles of the biosphere. Current research shows that microorganisms are involved in the formation of stalactites, moonmilks, and other mineral formations. However, the interactions of microbes with the air–water–rock interfaces in subterranean ecosystems and the biological mechanisms by which microorganisms adjust to new environments or changes in their current environment are poorly understood.

This Research Topic includes 16 articles that provide some clues to understand the *Microbial roles in caves*, and describes a broad range of microbial activities in subsurface environments.

The global biogeochemical cycles, and particularly the carbon, nitrogen, sulfur, and phosphorus cycles, are essential for life in caves. Zhu et al. discussed in their review the dissolution and deposition of carbonate minerals, the roles of cave microbes in the C, N, S, and Fe cycles, and the production of bioactive compounds and antimicrobials by cave microorganisms. Of particular importance is the involvement of methane-oxidizing bacteria in the consumption of cave methane. Bogdan et al. studied the diversity and distribution of microbial communities in a Romanian cave with low anthropic impact. Interestingly, the cave was largely dominated by the phyla *Pseudomonadota* and *Actinomycetota*, the same phyla frequently found in disturbed caves. A study of the potential functional role of these communities revealed the presence of genes involved in the C and N cycles.

Mondini et al. investigated the total and active prokaryotic and eukaryotic communities of millennium-old ice accumulated in Scarisoara Cave, Romania. The initial microbial community on cave ice was dominated by potentially active *Bacteria*, with a minor presence of *Archaea* and a low relative abundance of *Eukaryota*. Heat shock cycles had a strong impact on the composition of the ice microbiome, leading to important decreases of the relative abundance of *Archaea* and *Eukaryota*, while the bacterial community appeared to be more stable, prevailing in the ice community exposed to temperature fluctuations. The ice community was dominated by copiotrophic taxa that were able to quickly use the carbon sources released after ice thawing, with the *Pseudomonadota* and *Bacteroidota* taxa prevailing after thermal stress. This article offers a glimpse of the environmental impacts of climate change that lead to glacier retreat.

The microbiomes of three types of caves: limestone, sulfidic, and volcanic so far frequently studied were compared by Turrini et al.. These caves have different genesis, rock, and chemical composition which can determine diverse colonization patterns. A literature search identified the most prevalent bacterial taxa and the authors discussed the microbial diversity and their functional roles in the three cave types. Limestone and volcanic caves presented *Pseudomonadota* and *Actinomycetota* as primary colonizers, while sulfidic caves revealed the predominance of sulfur-oxidizing *Campylobacterota*. Good practices in future cave microbiome studies were discussed.

Prescott et al. studied the ecological drivers that structure the diversity and assemblies of the bacterial community in volcanic ecosystems in Hawai'i and compared the oldest lava tubes (500–800 years) to the more variable and extreme conditions of the younger geothermally active caves and fumaroles (< 400 years old). Data showed that lava caves and geothermal sites harbor unique microbial communities, with very little overlap between caves or sites. Additionally, older lava tubes hosted greater phylogenetic diversity than geothermally active or younger sites. Most ASVs were not able to be assigned to a named genus or species; therefore, volcanic caves and fumaroles represent underexplored ecosystems. In this context Gonzalez-Pimentel et al. investigated the microbial communities of volcanic caves in La Palma Island (Canary Islands, Spain) and described a new species of *Streptomyces, S. benahoarensis*, isolated from two different samples, a speleothem and a microbial mat on the walls of *Fuente de la Canaria* lava tube. The genes predicted involved in antimicrobial mechanisms for resistance and biosynthesis of these two strains emphasized this *Streptomyces* as a biological reservoir for bioactive compounds, both described and not discovered yet.

Castañar Cave (Caceres, Spain) is an extreme environment with very high concentrations of radon (^222^Rn) in air with an annual average >30 kBq/m^3^. In 2008, a vomit caused a fungal outbreak that was initiated by *Mucor circinelloides* and *Neocosmospora solani* and contaminated sediments. The cave sediments were cleaned and visits resumed in 2014. Martin-Pozas et al. analyzed the fungal community in the cave after 12 years of the fungal outbreak and the prevalence and spatio-temporal evolution of the fungi caused by the vomit over the years under the conditions of relative isolation and high radiation that characterize this cave. The occurrence of *N. solani* in all samplings from 2008 to 2020 is notable, as it has been widely reported in relation to outbreaks in other caves. Fungi previously reported in highly radioactive environments were also found in Castañar Cave, but the effect of high ^222^Rn on these fungi was not conclusive because the diversity was similar to that found in other caves with relatively low concentrations of ^222^Rn.

Fungal outbreaks also occur in other subsurface environments, as exemplified in the Roman Catacombs. De Leo et al. investigated the sudden fungal outbreak that occurred after 1 year of restoration treatment in the Catacombs of SS. Marcellino and Pietro in Rome (Italy). The restored marble pieces were colonized by a complex fungal biofilm consisting mainly of *Coniophora* sp. and other genera, such as *Hypomyces, Purpureocillium, Acremonium, Penicillium*, and *Alternaria*, many of which are well known for the biodeterioration of stone surfaces. The article features the first finding of a strain of the genus *Coniophora* (order *Boletales*), reported as one of the causes of wood wet-rot decay, in association with the evident phenomena of stone biodeterioration.

Ghezzi et al. reported that Imawarì Yeuta Cave (Venezuela) is composed of 98% silica in the form of α-quartz and minor amounts of amorphous silica. Microbial communities that inhabit caves in quartz-rich rocks are poorly known. A set of 19 biofilm samples in water ponds, quartzite host rocks, sediments and speleothems on pavement, walls, and ceiling at different sites within the cave was classified into three groups according to the water content: < 89%, 4%−20% and < 1%, and the dominant groups in each case were *Pseudomonadota* (*Gammaproteobacteria*), *Acidobacteriota*, and *Actinobacteriota*, respectively. Oligotrophy probably in association with the geochemistry of silica/quartz low pH buffering activity and alternative energy sources led to colonization of specific silica-associated microorganisms.

Wang et al. hypothesize that microbial communities living in cave rocks vary with zones, and mineral substrates contribute significantly to the variation. To test the hypothesis, the authors collected weathered rock samples from the entrance to the end in the Heshang Cave, China. The rocks were mainly composed of dolomite, calcite, Mg-calcite, quartz, and phosphate minerals (hydroxyapatite and fluorapatite). The bacterial communities were significantly affected by hydroxyapatite, and fluorapatite positively impacted the fungal communities, while the co-occurrence network showed that the bacterial and fungal communities formed a close organization through cooperation among different species. The effect of phosphate-rich deposits on the bacterial community in Muierilor Cave, Romania, was also studied by Haidău et al.. They found genera involved in the P, N, Fe, and Mn cycles, as such elements are usually found in guano, located in the upper level of the cave. Several genera were related to bats and guano, while others were related to humans derived from the impact of visits.

Microbially induced calcite precipitation (MICP) is defined as the formation of carbonate minerals from a solution due to the presence of cells, microbial products, or metabolic activity. Precipitation can be due mainly to modulation of the environmental pH, nucleation sites on cell surfaces, or by the action of enzymatically driven processes involving carbonic anhydrase, urease, etc. Koning et al. studied the MICP potential of cave bacteria isolated from the Iron Curtain Cave in Canada. Ninety-nine bacterial strains were isolated from popcorn and soda straw speleothems. These isolates were screened for urease enzymatic activity, with 11 candidates found to be positive for urease, including *Sphingobacterium* sp. and *Pseudarthrobacter* sp. which were found to produce the highest crystal production with varying morphologies. *Pseudarthrobacter* sp. encoded a single and complete urease pathway, whereas *Sphingobacterium* sp. showed two urease pathways encoded in the genome, with an unknown gene in the middle of the sequence.

Anglés et al. reported the formation of biospeleothems during the early Holocene in caves from the Uyuni Salar, Bolivia. The caves show bizarre speleothems framed by large fungal buildings (>1 m) covering the older mineralized structures of algae. The abundance and size of the preserved fungal structures suggest that they were sustained by a constant supply of organic matter and stable hydrological activity. The analysis of the lipids recorded in the samples also provided some insight into the paleoenvironmental conditions accompanying the formation of biospeleothems. It is worth noting the appearance of biomarkers from cyanobacteria and eukaryotes. The authors stated that the search for biomarkers in caves can answer questions about the limits of life and allow the recognition of the geochemical signatures of life.

In recent years, much attention has been paid to fungal infections that affect amphibians. Zalar et al. studied the cultivable skin mycobiota of healthy and diseased *Proteus anguinus*, the blind cave salamander, endemic to the Dinaric Karst, Slovenia, one of the priority species of the EU in need of strict protection. Symptomatic animals were colonized by a variety of fungal species, most of them represented by a single isolate, including genera known for their involvement in chromomycosis, phaeohyphomycosis, zygomycosis, and saprolegniosis in amphibians: *Acremonium, Aspergillus, Cladosporium, Exophiala, Fusarium, Mucor, Ochroconis, Phialophora, Penicillium*, and *Saprolegnia*. The article represents the first comprehensive report on the cultured skin mycobiome of this unique amphibian in nature and in captivity, with an emphasis on potentially pathogenic fungi and oomycetes.

Diverse gut bacteria are potentially involved in many physiological processes of insects, which contribute to the adaptation of host insects to the environment. Dong et al. studied the gut microbiota of the orthopteran *Diestrammena japanica*, a keystone species in the karst cave in China, and reported that individuals of different light strengths along the cave exhibit different morphological features. The gut bacteria of *D. japonica* exhibit low diversity but strong cooperation interactions in the dark region. These results indicated that intestinal bacteria may help *D. japanica* adapt to the poor nutrient cave environment.

Electrical lighting enhances the growth of photosynthetic communities known as lampenflora in cave entrances and speleothems. Djebaili et al. screened the cyanobacteria isolated from green biofilms in Stiffe caves, Italy, for the production of poly-β-hydroxybutyrate (PHB). The high production of PHB could be related to the lack of light for an extended period of closure in caves linked to COVID-19, as the accumulation of PHB allows coping with unfavorable environmental conditions.

This selection of articles should be viewed as a snapshot of the multiple biogeochemical processes and provide evidence that microbes play significant roles in caves.

## Author contributions

VJ: Writing—original draft, Writing—review & editing. DN: Writing—original draft, Writing—review & editing. CS-J: Writing—original draft, Writing—review & editing.

